# Hyperuricemia exacerbates abdominal aortic aneurysm formation through the URAT1/ERK/MMP-9 signaling pathway

**DOI:** 10.1186/s12872-022-03012-x

**Published:** 2023-01-30

**Authors:** Jen-Chun Wang, Shih-Hung Tsai, Hsiao-Ya Tsai, Shing-Jong Lin, Po-Hsun Huang

**Affiliations:** 1 Department of Emergency Medicine, Tri-Service General Hospital, National Defense Medical Center, No. 325, Sec. 2, Cheng-Kung Road, Neihu Dist., Taipei City, 114 Taiwan; 2 Institute of Clinical Medicine, National Yang-Ming University, Taipei, 112 Taiwan; 3 Institute of Clinical Medicine, National Yang Ming Chiao Tung University, Taipei, 112 Taiwan; 4 Division of Cardiology, Department of Internal Medicine, Taipei Veterans General Hospital, No. 201, Sec. 2, Shih-Pai Road., Taipei, Taiwan; 5 Cardiovascular Research Center, National Yang-Ming University, Taipei, Taiwan; 6 Department of Medical Research and Education, Taipei Veterans General Hospital, Taipei, Taiwan; 7 Department of Critical Care Medicine, Taipei Veterans General Hospital, Taipei, Taiwan

**Keywords:** Hyperuricemia, Uric acid, Urate transporter 1, Matrix metalloproteinase, Abdominal aortic aneurysm

## Abstract

**Objective:**

Previous studies have revealed associations between hyperuricemia and microvascular diseases, but the association between hyperuricemia and abdominal aortic aneurysm (AAA) remains unclear. The aim of this study was to elucidate the pathogenesis and prove the relationship between AAA and hyperuricemia.

**Methods:**

A retrospective study was performed to validate the growth rates of AAA in humans with different serum uric acid levels. A murine model of angiotensin II-induced AAA was used to assess the effects of hyperuricemia on AAA growth in vivo, and human aortic smooth muscle cells (HASMCs) were used to study the pathways involved in these effects in vitro.

**Results:**

We analyzed data from 107 AAA patients and found that patients with serum uric acid levels above 9 mg/dl had higher AAA growth rates than patients with serum uric acid levels between 4 and 7.9 mg/dl. In vivo, induction of hyperuricemia increased the incidence of AAA formation and the abdominal aortic diameter in mice. The hyperuricemic mice exhibited higher levels of urate transporter 1 (URAT1) expression, phospho-extracellular signal-regulated kinase (p-ERK)1/2 expression, reactive oxygen species (ROS) levels and matrix metalloproteinase (MMP)-9 expression in the abdominal aorta than the control mice. Soluble uric acid increased the expression of URAT1, p-ERK1/2, and MMP-9 and the levels of ROS in HASMCs in vitro.

**Conclusions:**

We have provided human evidence that hyperuricemia exacerbates AAA formation. In addition, our murine experimental evidence suggests that hyperuricemia exacerbates AAA formation and reveals that the URAT1/ERK1/2/ROS/MMP-9 pathway is among the pathways activated by uric acid in HASMCs.

**Supplementary Information:**

The online version contains supplementary material available at 10.1186/s12872-022-03012-x.

## Introduction

The main risk factors associated with abdominal aortic aneurysm (AAA) expansion and rupture include a large aneurysm diameter, a rapid rate of aortic diameter expansion, current smoking, and sex. AAA affects males to females at a ratio of 4:1, but females tend to have an increased risk of rupture and tend to experience rupture at smaller diameters [1, 2]. The formation of AAA is histologically distinct from atherosclerosis. Unlike atherosclerotic changes, which are limited to the inner layers of the aortic wall, AAA is characterized by transmural inflammatory changes. Specifically, the pathological features of AAA involve transmural inflammatory changes, smooth muscle cell damage, degradation of the matrix of the media layer of the aorta and abnormal collagen remodeling and cross-linking [3, 4]. The molecular pathways and molecules involved in AAA initiation and progression include the mitogen-activated protein kinase pathway, nuclear factor kappa-light-chain-enhancer of activated B-cell inflammatory pathways, matrix metalloproteinases (MMPs), and reactive oxygen species (ROS) [5–7]. MMPs play critical roles in degrading the collagen and elastin in the media layer of the aorta and in subsequent AAA formation and progression [8].

Several epidemiological studies have revealed an association among hyperuricemia, atherosclerosis and cardiovascular diseases (CVDs) [9]. Hyperuricemia independently predicts the development of hypertension, stroke and heart failure [10]. Soluble uric acid can mediate the generation of free radicals and function as a pro-oxidant. Hyperuricemia also promotes inflammation and the expression of MMP-9 in individuals with gouty arthritis [11]. At the cellular level, urate transporter 1 (URAT1) is considered an organic anion transporter [12]. URAT1 has also been found in the cell membranes of human aortic smooth muscle cells (HASMCs) and may provide a mechanism through which uric acid enters HASMCs [13]. Uric acid can increase superoxide anion levels in vascular smooth muscle cells, and ROS play key roles in the regulation of MMPs and the induction of vascular smooth muscle cell apoptosis [14]. However, the relationship between ROS and hyperuricemia in rodent studies remains controversial [8, 15]. Therefore, the roles of hyperuricemia-induced vascular inflammation and ROS in the pathogenesis of AAA formation remain unclear.

Taken together, the abovementioned lines of evidence indicate that hyperuricemia is associated with several AAA-related comorbidities and molecular mechanisms. However, the association between AAA and hyperuricemia remains undefined. We hypothesized that hyperuricemia could promote AAA formation and acceleration through vascular inflammation and ROS production. Thus, we aimed to perform proof-of-concept animal studies to mechanistically confirm the association between hyperuricemia and AAA and the roles of hyperuricemia in the pathogenesis of AAA.

## Materials and methods

For the human data, we used a single-center retrospective study to determine the correlation between hyperuricemia and the AAA expansion rate. Furthermore, we used an angiotensin II (Ang II)-induced AAA hyperlipidemic mouse model to confirm the causal relationship between hyperuricemia and AAA. We conducted in vitro experiments using HASMCs to elucidate the molecular mechanism.

### Human clinical data

To determine whether hyperuricemia aggravates human AAA growth, we analyzed the serum uric acid levels and abdominal aortic diameter expansion rates of hospitalized patients in a tertiary medical center. During a 5-year period, 279 consecutive patients with a diagnosis of AAA were admitted to Tri-Service General Hospital, Taiwan, regardless of the reasons for hospitalization. All patients underwent computer tomography scans or abdominal sonography for confirmation and measurement of the size of the abdominal aorta. Patients who had missing data on serum uric acid levels, a lack of follow-up images after the first imaging round revealed AAA, and a history of surgical intervention for AAA were excluded. We also excluded patients who received the first and last computed tomography scans or abdominal sonography within fewer than 3 months. A total of 107 patients were included. The patients were divided into seven groups according to their serum uric acid levels, as follows: < 4 mg/dl, 4–4.9 mg/dl, 5–5.9 mg/dl, 6–6.9 mg/dl, 7–7.9 mg/dl, 8–8.9 mg/dl and ≥ 9 mg/dl. The expansion rate of the diameter of the abdominal aorta was calculated in centimeters per year. The study was approved by the Institutional Review Board of Tri-Service General Hospital at the National Defense Medical Center in Taipei, Taiwan (TSGHIRB No. 1-105-05-176 and No. 2-106-05-097).

### Animal and in vitro experiments

#### Human cells and reagents

HASMCs were purchased from ScienCell Research Laboratories (#6110) and maintained in M231 medium (#M231500) supplemented with supplemental medium (#S00725) and 5% fetal bovine serum (#10437028). The cells were incubated at 37 °C in an atmosphere with 5% CO_2_. Ang II (Sigma–Aldrich, A9525), uric acid (Sigma–Aldrich, U0881), *N*-acetylcysteine (NAC, Sigma–Aldrich, A7520), and PD98059 (Sigma–Aldrich, P215). Short interfering RNA targeting URAT1 (siURAT1, sc-96373) and a scrambled sequence were used for the in vitro experiments. The interfering RNA and all culture media were purchased from Santa Cruz. HASMCs were transfected with 10 nM siURAT1 (sc-96373) or 10 nM control siRNA (Life Technologies, #4464058). siURAT1 was complexed with a transfection reagent (Bio-Rad, siLentfect 1703361) according to the manufacturer’s guidelines. The HASMCs were treated with 10 μM PD98059 dissolved in DMSO and 1 mM NAC dissolved in double-distilled H_2_O.

### Animal experiments

Eight- to ten-week-old male apolipoprotein E (ApoE)-knockout (KO) mice on a C57BL/6J background were obtained from Jackson Laboratories. Hyperuricemia was induced in the mice as previously described [16]. Briefly, the mice were administered an intraperitoneal injection of potassium oxonate (200 mg/kg; # 156124, Sigma Chemical Co., St. Louis, MO, USA) at 08:00 for a total of 8 weeks and fed a uric acid-enriched diet consisting of normal chow supplemented with 2.5% uric acid [17]. To establish the Ang II-induced AAA model, ALZET osmotic minipumps (model 2004; ALZET Scientific Products, Mountain View, CA, USA) were implanted into the mice at the beginning of the 5th week after the start of hyperuricemia induction. The pumps delivered 1000 ng/kg/min Ang II (A9525, Sigma Chemical CO.) for 4 weeks as previously described [18]. The mice were randomly assigned to 1 of the following 4 treatment groups: (1) the control group (in which the mice were treated with the same volume of saline); (2) the hyperuricemia group (in which\hyperuricemia was induced in the mice as described above); (3) the Ang II group (in which Ang II-induced AAA model mice were established as described above); and (4) the hyperuricemia + Ang II group. All of the mice were given ad libitum access to food and water and were maintained in microisolator cages under a 12-h day/night cycle. The body weight of the animals was monitored during treatment for assessment of side effects. At the end of the 8-week study, the mice were euthanized by exsanguination under anesthesia. Blood was drawn from the right heart ventricle for analysis. The aorta was exposed under a dissecting microscope, and the periadventitial tissue was removed from the aortic wall. The gross appearance of the aorta was digitally photographed, and the maximal external diameter of the suprarenal aorta was measured using imaging processing software (ImageJ). The development of an aortic aneurysm was defined by an aortic diameter growth greater than 50%, as previously described [19]. After carefully removing the periaortic soft tissue, the entire aorta was perfused with saline and excised. All experimental protocols and procedures were approved by the institutional animal care committee of the National Defense Medical Center (Taipei, Taiwan) and complied with the Guide for the Care and Use of Laboratory Animals published by the US National Institutes of Health (8th edition, 2011) [20].

Whole blood was collected from the mice and allowed to stand at room temperature for 30 min. The serum was separated by centrifugation at 3000×*g* and 4 °C for 15 min and maintained at − 80 °C until analysis. We measured the serum samples using biochemical slides with an automated Clinical Chemistry Analyzer (FUJI DRI-CHEM 4000i). Aortas that were perfusion-fixed in formalin prior to harvest were embedded in paraffin or frozen at − 80 °C, cut into cross-sections (5 μm) and stained with hematoxylin and eosin and Verhoeff-Van Gieson (VVG) for observation of elastin. The severity of elastin degradation was semiquantified as previously described with the following scale: grade 1, elastin degradation in < 25% of the entire area; grade 2, elastin degradation in 25–50% of the entire area; grade 3, elastin degradation in 50–75% of the entire area; and grade 4, aortic rupture or elastin degradation in > 75% of the entire area [21].

### Immunoblotting

Cell culture dishes were placed on ice, and the cells were washed with ice-cold PBS. The PBS was aspirated, and ice-cold lysis buffer was added. The adherent cells were scraped, and the cell suspension was transferred into a microcentrifuge tube. After centrifugation, the supernatant was aspirated as the protein lysate. The protein lysate (30 μg) was subjected to SDS–PAGE, and the proteins were then electrophoretically transferred onto a PVDF membrane. The membranes were probed with monoclonal antibodies against URAT1 (Proteintech, 14937-1-AP), MMP-9 (Cell Signaling Technology (CST), #13667), phospho-ERK1/2 (CST, #9106), ERK1/2 (CST, #4695), and α-tubulin (CST, #2144). The bands were visualized using chemiluminescence detection reagents. The immunoblots of the target proteins were quantified by densitometry, and the quantities are expressed relative to the expression of an internal control (for nonphosphorylated proteins) or to total protein expression (for phosphorylated proteins). The densitometric analysis was conducted using imaging processing software (Multi Gauge, Fujifilm), and the data are expressed as fold changes relative to controls.

### Measurement of ROS

ROS measurements were performed using an OxiSelect™ In Vitro ROS/RNS Assay Kit (STA-347, Cell Biolabs, Inc., San Diego, CA, USA) according to the manufacturer’s recommendations. This in vitro assay measures total ROS/reactive nitrogen species (RNS) free radical activity. Unknown ROS or RNS samples or standards were added to the wells with a catalyst that helped accelerate the oxidative reaction. The samples were measured fluorometrically against hydrogen peroxide. The free radical content in the samples was determined through comparison with a hydrogen peroxide standard curve. In brief, the cell lysate homogenates were stained with 2′,7′-dichlorofluorescein diacetate, which is oxidized by ROS to form fluorescent 2′,7′-dichlorofluorescein. The samples were loaded into 96-well black plates for 30 min at 37 °C, and the relative fluorescence units were measured with an excitation wavelength of 488 nm and an emission wavelength of 535 nm using a fluorescence microplate reader.

### Real-time polymerase chain reaction (RT-PCR)

Total RNA was isolated with TRIzol reagent (Invitrogen, 15596018), and cDNA was synthesized with a reverse transcription kit (Thermo Fisher Scientific, K1691). Real-time PCR was performed in duplicate using URAT1, GLUT9 and U6 primers with SYBR qPCR mix (Thermo Fisher Scientific, 4385612) according to the manufacturer’s protocol.

### Statistical analysis

All experiments were performed independently at least 3 times, and all continuous variables are presented as the means ± standard errors of the means (SEMs). Comparisons between two groups were performed using Student’s t test. The normality of the data was analyzed using the Shapiro–Wilk test. Data from multiple-group comparisons and nonnormally distributed data were analyzed using the Kruskal–Wallis test. Dunn’s multiple-comparison post hoc test was applied to correct for multiple comparisons. Data from multiple groups were analyzed with one-way ANOVA followed by Tukey’s multiple comparison test. G*Power version 3.1.9.7 was used for the power analysis. The sample size of the murine experiments was calculated based on effect sizes using the results from a previous study. A post hoc power analysis was performed with the final samples. The power analysis using an F test suggested a power of 0.97 with an alpha of 0.05 and an effect size of 0.75. A power of 0.95 for the human study. The target protein expression measured by immunoblotting was determined via densitometry and is expressed relative to the expression of an internal control (for nonphosphorylated proteins) or to the total protein expression (for phosphorylated proteins). A *P* value less than 0.05 was defined to indicate statistical significance. The analyses were performed using a statistical software package (SPSS version 22.0 for Windows; SPSS Inc., Chicago, IL, USA) and GraphPad software.

## Results

### Hyperuricemia exacerbates the expansion rates of AAA in humans

**Fig. 1:**
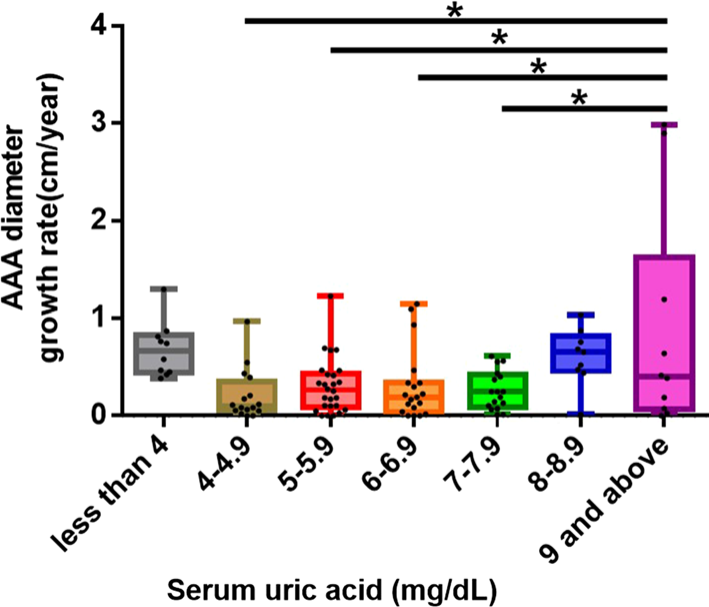
Hyperuricemia is associated with abdominal aortic diameter expansion. Hyperuricemia increases the rate of abdominal aortic diameter expansion. Patients with serum uric acid levels ≥ 9 mg/dl have more rapid abdominal aortic diameter expansion rates than patients with serum uric acid levels between 4 and 7.9 mg/dl. The upper and lower whiskers show the maximal and minimal values, respectively, and the upper, middle and lower horizontal lines in the box represent the upper, middle and lower quartiles, respectively. *P < 0.05

To determine whether hyperuricemia aggravates human AAA growth, we enrolled 107 patients who had AAA in a tertiary medical center. The follow-up time ranged from 129 to 4505 days, and the mean follow-up duration was 1275 days (3.49 years). Compared to patients who had serum uric acid levels between 4.0 and 7.9 mg/dl, patients who had serum uric acid levels of 9 mg/dl and above had higher rates of abdominal aortic diameter expansion (4–4.9 mg/dl, 0.2 cm/year, n = 16; 5–5.9 mg/dl, 0.3 cm/year, n = 26; 6–6.9 mg/dl, 0.3 cm/year, n = 19; 7–7.9 mg/dl, 0.26 cm/year, n = 17; 8–8.9 mg/dl, 0.6 cm/year, n = 9; 9 mg/dl and above, 0.88 cm/year, n = 10, *P* < 0.05). Patients who had serum uric acid levels less than 4 mg/dl did not have significantly different rates of aortic diameter expansion from patients in the other groups (0.68 cm/year, n = 10) (Fig. 1).

### Hyperuricemia aggravates Ang II-induced AAA formation in ApoE-KO mice

**Fig. 2:**
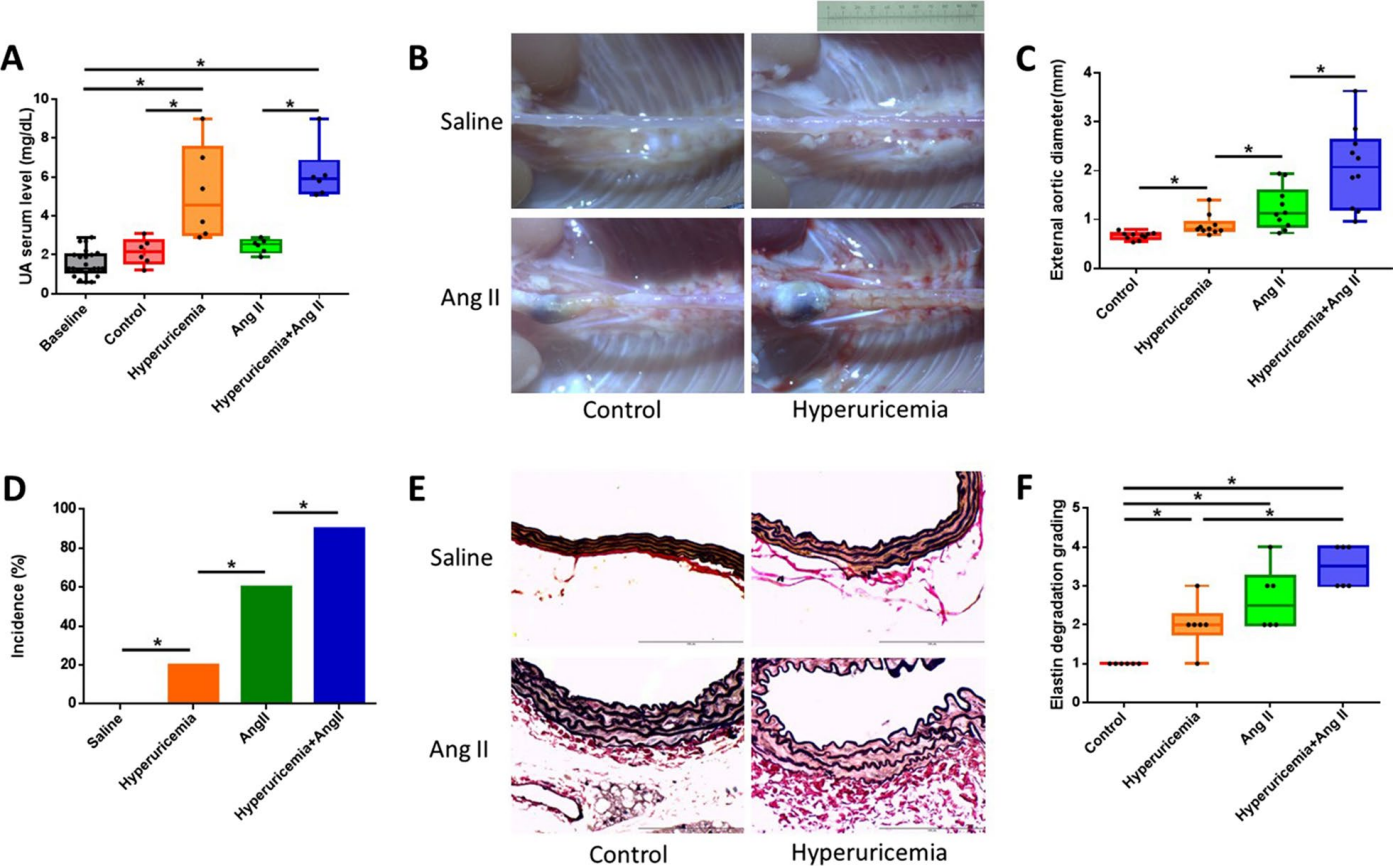
Hyperuricemia aggravates AAA formation in mice. Hyperuricemia markedly enhanced the aortic aneurysm severity and increased the incidence of AAA formation. Hyperuricemic mice exhibited more advanced elastin degradation in the abdominal aorta than control mice. **A** Serum uric acid levels at baseline and at the endpoint of the experiment. **B** Representative photographs of macroscopic features of aneurysms in male ApoE-KO mice. Control, saline infusion; hyperuricemia, potassium oxonate injection, uric acid in the diet plus saline infusion; Ang II, Ang II (1000 ng/kg per minute) infusion for 4 weeks; hyperuricemia + Ang II, potassium oxonate injection, uric acid in the diet plus saline infusion and Ang II infusion. Scale bar = 10 mm. **C** Maximum external diameter of the abdominal aorta. **D** Incidence rate of AAA (percentage). **E** Representative VVG staining of the abdominal aorta. Scale bar = 100 μm. **F** Statistical analysis of elastin degradation. ApoE-KO: apolipoprotein E-knockout; Ang II: angiotensin II; VVG: Verhoeff-Van Gieson. The upper and lower whiskers show the maximal and minimal values, respectively, and the upper, middle and lower horizontal lines in the box represent the upper, middle and lower quartiles, respectively. *P < 0.05

The mean baseline serum uric acid level of all the experimental mice before the experiment was 1.53 mg/dl. The uric acid- and potassium oxonate-treated mice developed hyperuricemia, and their serum uric acid levels were significantly different from those of the untreated mice (control, 2.15 mg/dl; hyperuricemia, 5.18 mg/dl; Ang II, 2.46 mg/dl; and hyperuricemia + Ang II, 6.2 mg/dl; *P* < 0.01; Fig. 2A). Consistent with the findings of previous studies, continuous infusion of ApoE-KO mice with Ang II for 4 weeks induced AAA development in the suprarenal aorta, as shown in Fig. 2B. Hyperuricemia markedly enhanced the aortic aneurysm severity, as reflected by the maximal abdominal aortic diameter (control, 0.66 mm; hyperuricemia, 0.88 mm; Ang II, 1.22 mm; and hyperuricemia + Ang II, 2.07 mm; n = 10, respectively; *P* < 0.05; Fig. 2C). Using a diameter expansion of 50% compared with the control as the definition for AAA, hyperuricemia also significantly increased the incidence of AAA formation (control, 0% (0/10); hyperuricemia, 20% (2/10); Ang II, 60% (6/10); and hyperuricemia + Ang II, 90% (9/10); n = 10, *P* < 0.05, Fig. 2D). VVG staining showed that the hyperuricemic mice exhibited more advanced elastin degradation in the abdominal aorta than the control mice (semiquantification of each group, *P* < 0.01, Fig. 2E, F).

### Hyperuricemia amplifies p-ERK1/2 and MMP-9 expression in the suprarenal aortas of Ang II-infused ApoE-KO mice

**Fig. 3:**
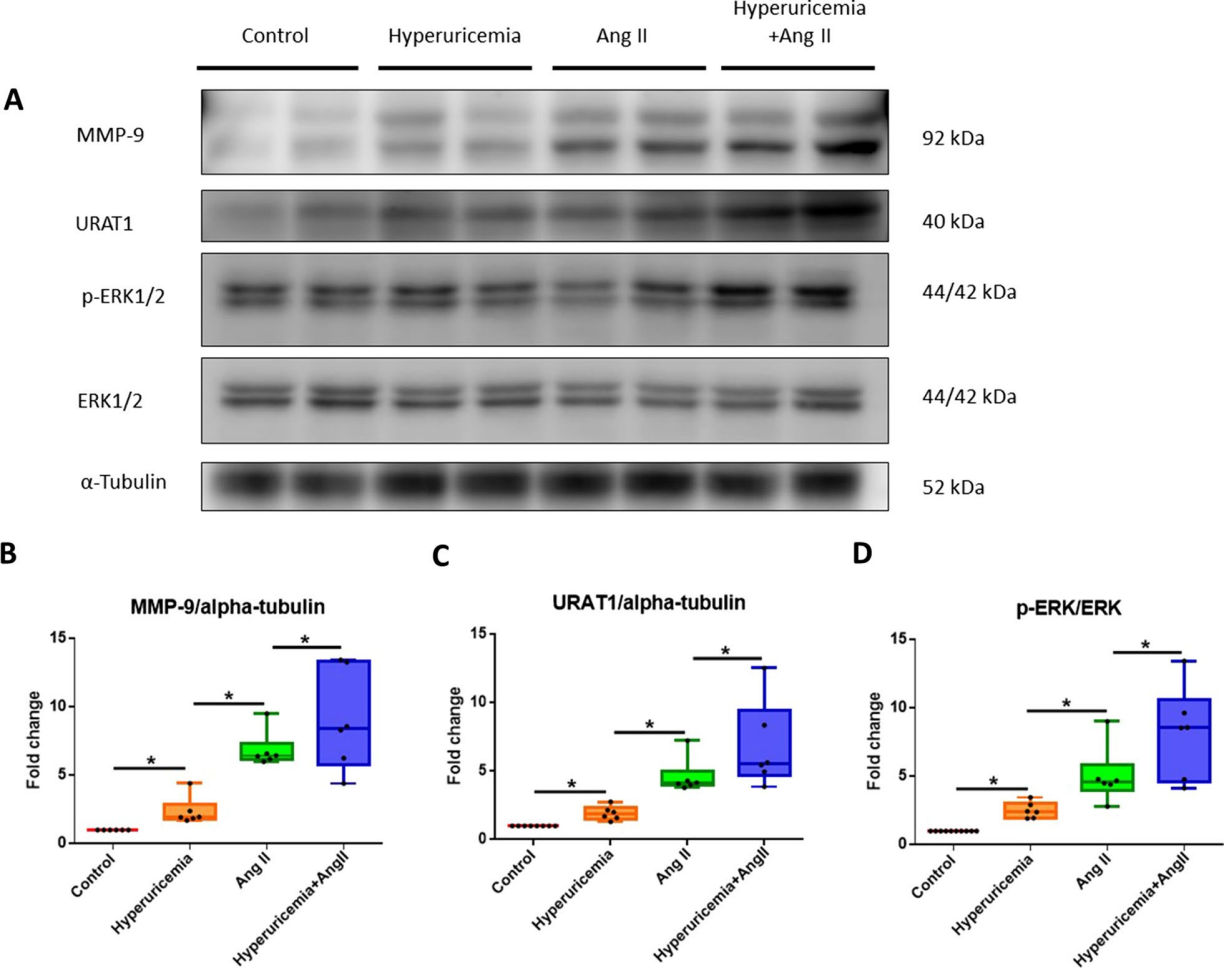
Hyperuricemia promotes increases in MMP-9, URAT1 and p-ERK 1/2 protein abundance in the abdominal aorta in mice. **A** Representative Western blot analysis and quantification of MMP-9, URAT1, p-ERK1/2 and ERK1/2 protein expression in the aortas of ApoE-KO mice with hyperuricemia or treated with Ang II. Hyperuricemia alone enhanced MMP-9, URAT1 and p-ERK1/2 expression compared with the control level. Ang II and hyperuricemia + Ang II also increased the expression abundance of MMP-9, URAT1 and p-ERK1/2. **B** MMP-9 protein abundance. **C** URAT1 protein abundance. **D** p-ERK1/2 protein abundance. MMP: matrix metalloproteinase; URAT1: urate transporter 1; p-ERK 1/2: phospho-extracellular signal-regulated kinases 1/2; Ang II: angiotensin II. The upper and lower whiskers show the maximal and minimal values, respectively; and the upper, middle and lower horizontal lines in the box represent the upper, middle and lower quartiles, respectively. Original images of blots (Additional file 1). *P < 0.05

The administration of Ang II significantly increased the expression of p-ERK1/2 (5.04-fold, n = 6, *P* < 0.05) and MMP-9 (6.86-fold, n = 6, *P* < 0.05) in the abdominal aortas of ApoE-KO mice, which is consistent with observations from human and mouse aneurysmal tissue [22]. Ang II also increased the abundance of URAT1 (4.60-fold, n = 6, *P* < 0.05). Furthermore, the induction of hyperuricemia alone enhanced the abundance of MMP-9, URAT1, and p-ERK1/2 expression in the abdominal aorta in ApoE-KO mice compared with control mice (2.37-fold, n = 6, *P* < 0.05; 1.92-fold, n = 6, *P* < 0.05; and 2.51-fold, n = 6, *P* < 0.05). The mice in the hyperuricemia + Ang II group showed increased levels of MMP-9 (9.04-fold, n = 6, *P* < 0.05), URAT1 (6.8-fold, n = 6, *P* < 0.05), and p-ERK1/2 (8.19-fold, n = 6, *P* < 0.05) (Fig. 3).

### URAT1 and p-ERK1/2 mediate soluble uric acid-induced oxidation in HASMCs in vitro

**Fig. 4:**
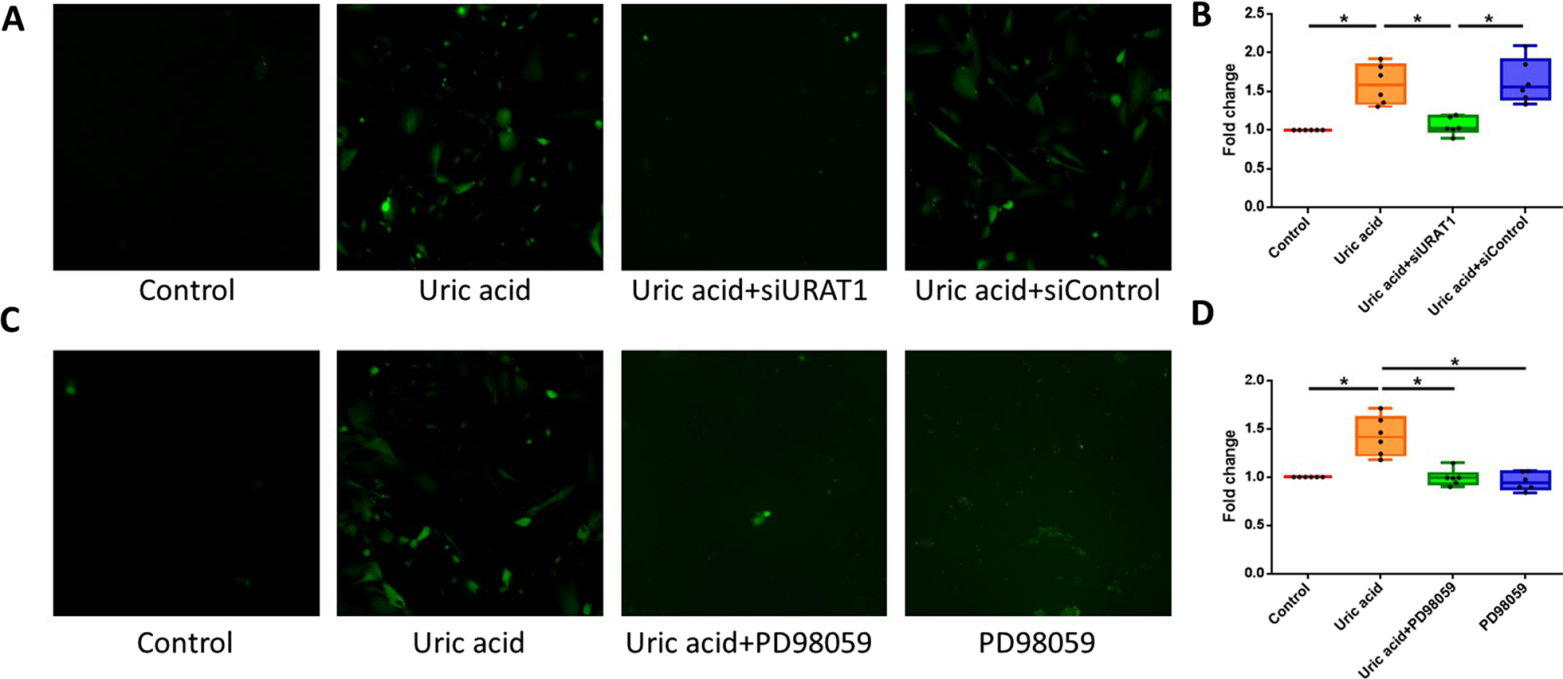
Soluble uric acid stimulates ROS production through URAT1 and p-ERK1/2 in HASMCs. The ROS levels in HASMCs were measured by chemiluminescence. The uric acid-induced production of ROS was mediated by p-ERK1/2 through URAT1 in HASMCs. **A** and **B** Uric acid stimulated ROS production in HASMCs, but transfection of URAT1 siRNA decreased the effect of uric acid on ROS production. **C** and **D** The MEK inhibitor PD98059 decreased the inducing effect of uric acid on ROS production. ROS: reactive oxygen species; URAT1: urate transporter 1; p-ERK1/2: phospho-extracellular signal-regulated kinase 1/2; HASMCs: human aortic smooth muscle cells; MEK: mitogen-activated protein kinase kinase. The upper and lower whiskers show the maximal and minimal values, respectively, and the upper, middle and lower horizontal lines in the box represent the upper, middle and lower quartiles, respectively. *P < 0.05

To further explore the mechanism underlying the induction of oxidation by uric acid, we analyzed the production of ROS in uric acid-treated HASMCs. Treatment with soluble uric acid (10 mg/dl) increased the generation of ROS in HASMCs by 1.59-fold compared with that obtained without treatment. Furthermore, a 1.51- or 1.43-fold reduction in the uric acid-induced production of ROS was observed in HASMCs transfected with URAT siRNA or an MEK inhibitor (PD98059) (*P* < 0.05). Taken together, these results revealed that the uric acid-induced production of ROS was mediated by p-ERK1/2 through URAT1 in HASMCs (Fig. 4).

### Soluble uric acid induces phosphorylation of ERK1/2 and ERK1/2 protein expression to increase MMP-9 levels and ROS production through URAT1 in HASMCs in vitro

**Fig. 5:**
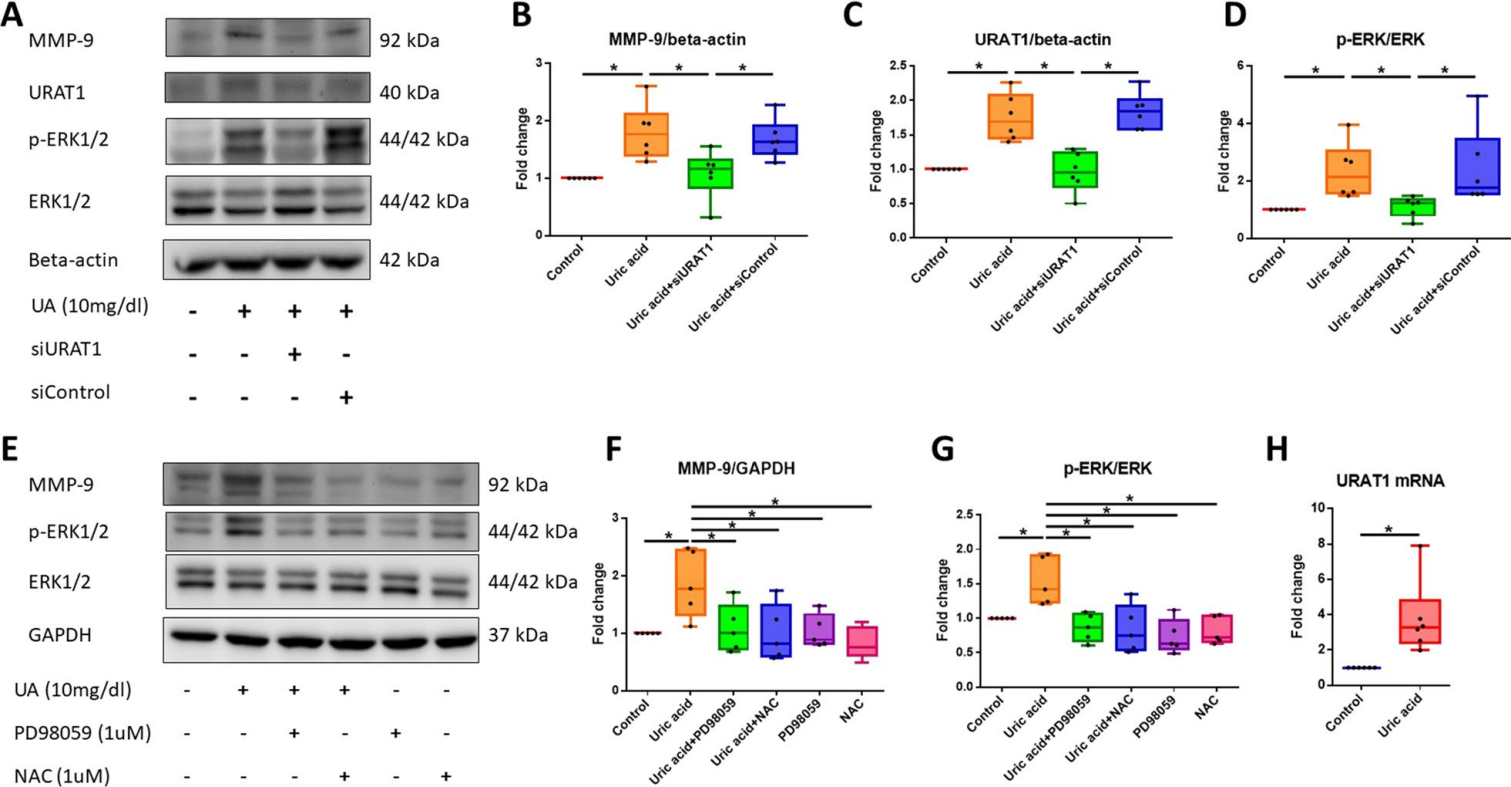
Soluble uric acid stimulates p-ERK1/2 and MMP9 production through URAT1 in HASMCs. **A** Protein abundance in cells that were stimulated or not stimulated with uric acid and transfected with siURAT1. **B** MMP-9 protein expression abundance. **C** URAT1 protein abundance. **D** p-ERK1/2 protein abundance. **E** Protein abundance in cells stimulated or not stimulated with uric acid, PD98059 or NAC. **F** MMP-9 protein expression abundance. **G** p-ERK protein abundance. **H** Fold changes in the relative mRNA expression of URAT1 between the uric acid-treated group and the control group. The Ct value of URAT1 obtained by RT–PCR and that of the internal control. p-ERK1/2: phospho-extracellular signal-regulated kinase 1/2; MMP: matrix metalloproteinase; URAT1: urate transporter 1; HASMCs: human aortic smooth muscle cells; NAC: N-acetylcysteine. The upper and lower whiskers show the maximal and minimal values, respectively, and the upper, middle and lower horizontal lines in the box represent the upper, middle and lower quartiles, respectively. Original images of blots (Additional file 1). *P < 0.05

To confirm that soluble uric acid-induced increases in MMP-9 protein levels are mediated by URAT1 and are involved in the p-ERK1/2 signaling pathways, we transfected siURAT1 (10 nM) and control siRNA (10 nM) into HASMCs before uric acid treatment. As shown in Fig. 5, the silencing of URAT1 attenuated the uric acid-induced increases in MMP-9 and URAT1 abundance compared with the abundance observed in the control uric acid-treated cells (1.81-fold, *P* < 0.05; 1.82-fold, *P* < 0.05) and attenuated the uric acid-induced phosphorylation of ERK1/2 compared with that found in the control uric acid-treated cells (2.11-fold, *P* < 0.05). The administration of a MEK inhibitor reduced the soluble uric acid-induced increase in the protein expression of MMP-9 (1.76-fold, *P* < 0.05) in HASMCs in vitro. To confirm the effect of ROS on the signaling pathway, we added NAC (1 μM) as an antioxidant agent. NAC attenuated the uric acid-induced increase in MMP-9 protein expression compared with that found in control uric acid-treated cells (1.87-fold, *P* < 0.05), but NAC did not attenuate the uric acid-induced phosphorylation of ERK1/2 compared with that found in the control uric acid-treated cells. These results indicate that MMP-9 is regulated by the URAT1, p-ERK1/2 and ROS pathways in HASMCs. We also determined the relative mRNA expression of URAT1 in HASMCs and found that the relative mRNA expression of URAT1 (3.78-fold, *P* < 0.05) was increased after treatment with soluble uric acid (Fig. 5).

## Discussion

In this study, we found that hyperuricemia was associated with the development and progression of AAA in a single medical center database. We further confirmed the association between AAA and hyperuricemia in a murine model of AAA. Mechanistically, we demonstrated a signaling pathway by which uric acid induced inflammation through URAT1-mediated ERK, ROS and MMP-9 production in HASMCs.

Previous studies have shown an association between hyperuricemia and microvascular disease. Hyperuricemia was identified as a risk factor for aortic disease-related death in a 3.8-year-long nationwide community-based cohort study in Japan [23]. We performed a study in a tertiary medical center in Taiwan. We revealed that AAA patients who had serum uric acid levels above 9 mg/dl had greater expansion rates of the abdominal aorta than those who had serum uric acid levels between 4 and 7.9 mg/dl. A previous study has indicated that serum uric acid levels ≥ 8 or < 4 mg/dL independently predict higher all-cause and CVD-related mortality in elderly patients [24]. Consistently, we also demonstrated a trend of U-shaped association between serum uric acid levels and AAA expansion rate.

We further confirmed these findings using an Ang II-induced AAA model. Previous studies have shown that the pathogenesis of AAA includes inflammation of the media layer of the aorta and degradation of elastin fibers. In addition, MMPs play key roles in AAA via degeneration of the abdominal aorta [8, 25]. We found that hyperuricemia itself increased the expansion of the diameter of the abdominal aorta, the incidence of AAA and the degradation of elastin fibers in the aorta in ApoE-KO mice. Unlike the effect of Ang II in the Ang II-induced AAA mouse model, which is well established, very few studies have shown that hyperuricemia alone can cause AAA formation in mice. In our study, mice with hyperuricemia alone that were not treated with Ang II presented increased aortic diameters, exacerbated elastin degradation, and elevated MMP-9 protein levels. Some mice with hyperuricemia alone presented expanded abdominal aortas; these findings were consistent with AAA based on the definition of a diameter expansion greater than 50%. Our data support the conclusion that hyperuricemia alone is an exacerbating factor of AAA formation in ApoE-KO mice. Hyperuricemia also enhanced Ang II-induced AAA formation, abdominal aortic diameter expansion and elastin fiber degradation, which suggests roles for hyperuricemia in the pathogenesis of AAA. In addition, we found that Ang II induced an increase in URAT1 protein expression in the mouse aorta. This finding is consistent with a previous report showing that losartan, an Ang II receptor blocker, can decrease the mRNA expression of URAT1 in HEK293 cells [26]. Mice with hyperuricemia presented higher protein levels of URAT1, p-ERK1/2 and MMP-9 in aortic tissue than mice with normal serum uric acid levels. This finding suggests that hyperuricemia may promote AAA and that the URAT1/ERK1/2/MMP-9 pathway is one of the possible pathogenic pathways.

Mechanistically, we found that silencing URAT1 attenuated the uric acid-induced activation of ERK and ROS production. URAT1 is reportedly transported to the cell membrane and may participate in a mechanism through which uric acid enters HASMCs [13]. Soluble uric acid can mediate the generation of free radicals and function as a pro-oxidant [9]. Hyperuricemia also promotes inflammation and the expression of MMP-9 in the synovial fluid of patients with gouty arthritis [11]. Our study demonstrates that soluble uric acid may activate MMP-9 protein expression in HASMCs through the URAT1, ROS and ERK1/2 pathways. Soluble uric acid can activate intracellular ROS production and ERK1/2 and MMP-9 phosphorylation in HASMCs. Furthermore, the effect of uric acid can be alleviated by transfection with URAT1 siRNA. In HASMCs, ROS and MMP-9 production was increased after treatment with soluble uric acid, and a MEK inhibitor significantly reversed this effect. In addition, the antioxidant NAC decreased MMP-9 production but did not significantly suppress the uric acid-induced phosphorylation of ERK1/2.

Our findings suggest that the molecular mechanism of soluble uric acid in HASMCs involves stimulation of the URAT1/ERK1/2/ROS/MMP-9 signaling pathway. Among the factors, MMP-9 protein expression is a key step in media layer degradation and subsequent AAA formation.

### Limitations

First, the sample size in our single-center retrospective study was limited. Further studies with large sample size and cofounder matching are warranted. Second, the continuous Ang II infusion-induced AAA model in hyperlipidemic mice recapitulates many major pathological features of AAA in humans, such as atherosclerosis, medial hypertrophy, macrophage accumulation in the external elastic lamina and thrombosis [26]. However, several features of this AAA model are not consistent with those of human AAAs, including the location (suprarenal vs. infrarenal) and severity of calcification [27, 28]. Third, serum uric acid affects HASMCs through URAT1. Loss-of-function tests involving inhibition of URAT1 in mouse experiments could provide more solid evidence. However, URAT1 is also an important participant in urate reabsorption regulation in the kidneys. Furthermore, URAT1- and VSMC-specific KO mice are needed to confirm the roles of URAT1 in the pathogenesis of AAA. Finally, our animal experiments were based on ApoE-KO mice. Further investigation regarding whether hyperuricemia in wild-type mice is capable of inducing AAA is warranted.

## Conclusion

In summary, we have shown an association between hyperuricemia and AAA in a human study and provided experimental evidence that hyperuricemia exacerbates AAA formation in a murine model. The URAT1/p-ERK1/2/ROS/MMP-9 pathway is one of the possible pathways activated by uric acid in HASMCs.

## Supplementary Information

**Additional file 1.** Original images of full-length blots of Figs. 3 and 5A, E. Please note some images of Fig. 3 are not full-length due to technical factors.

## Data Availability

All data generated or analyzed during this study are included in this published article.

## Abbreviations

AAA: Abdominal aortic aneurysm
Ang II: Angiotensin II
VVG: Verhoeff-Van Gieson
HASMCs: Human aortic smooth muscle cells
URAT1: Urate transporter 1
p-ERK: Phosphorylated extracellular signal-regulated kinase
ROS: Reactive oxygen species
MMP: Matrix metalloproteinase
ApoE-KO: Apolipoprotein E-knockout
MEK: Mitogen-activated protein kinase kinase
