# Supplementary material for: Hyperuricemia exacerbates abdominal aortic aneurysm formation through the URAT1/ERK/MMP-9 signaling pathway
Source: BMC Cardiovasc Disord. 2023 Jan 30;23:55. doi: 10.1186/s12872-022-03012-x (PMC9885634; doi:10.1186/s12872-022-03012-x)
Supplement: Supplementary file 1 — Additional file 1. Original images of full-length blots of Figs. 3 and 5A, E. Please note some images of Fig. 3 are not full-length due to technical factors. [file 12872_2022_3012_MOESM1_ESM.pptx]

## Slide 1
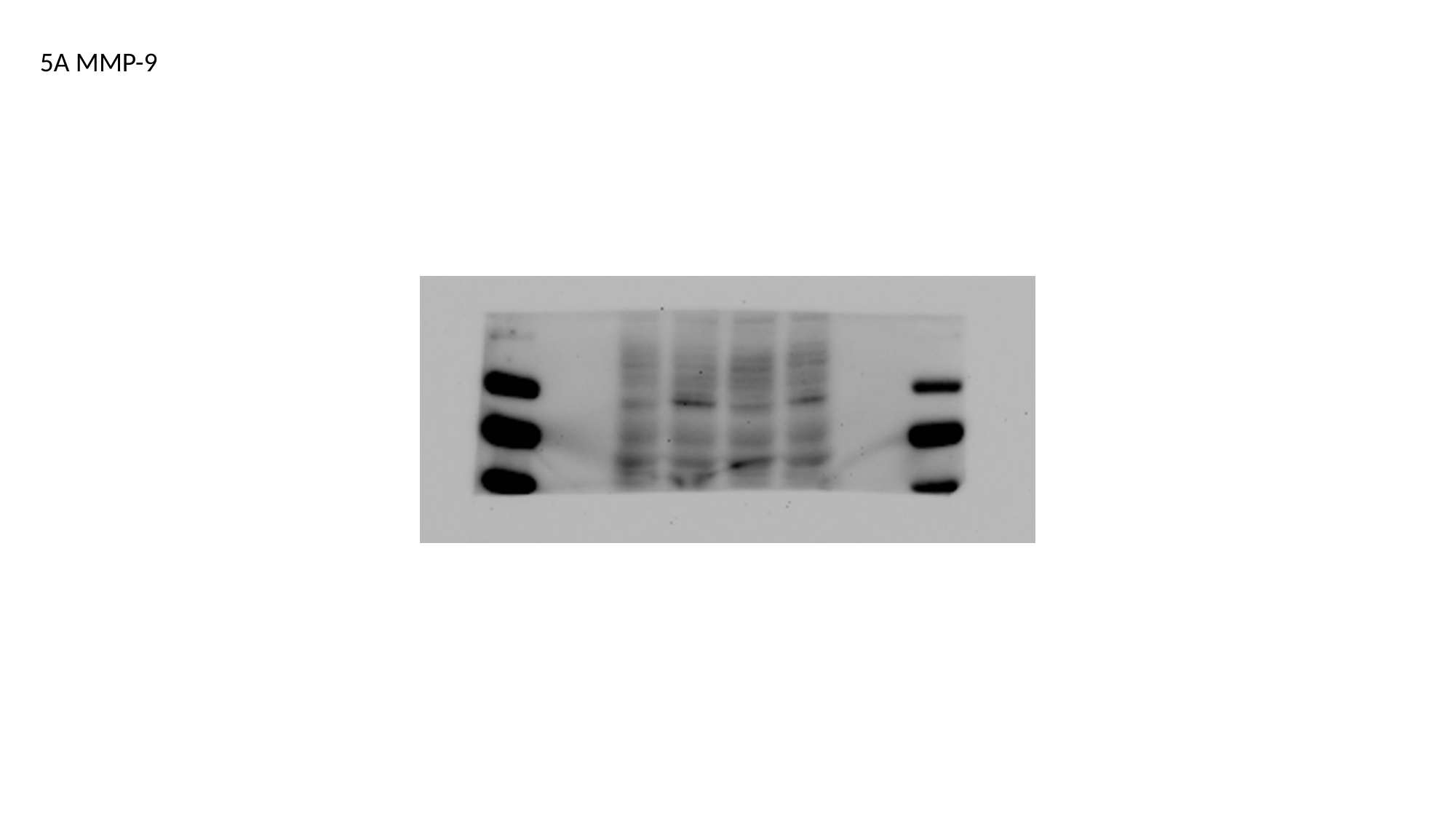

5A MMP-9

## Slide 2
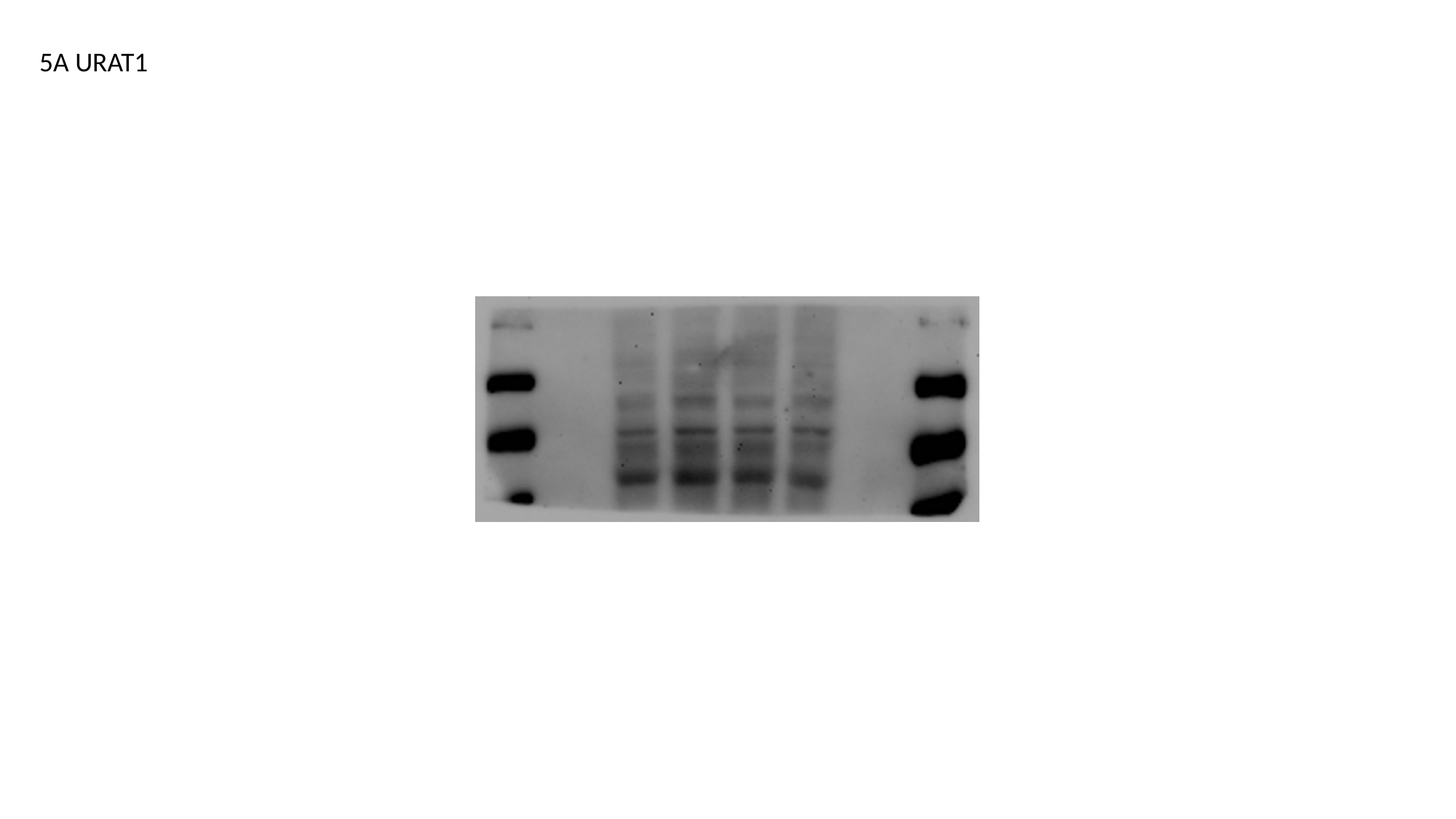

5A URAT1

## Slide 3
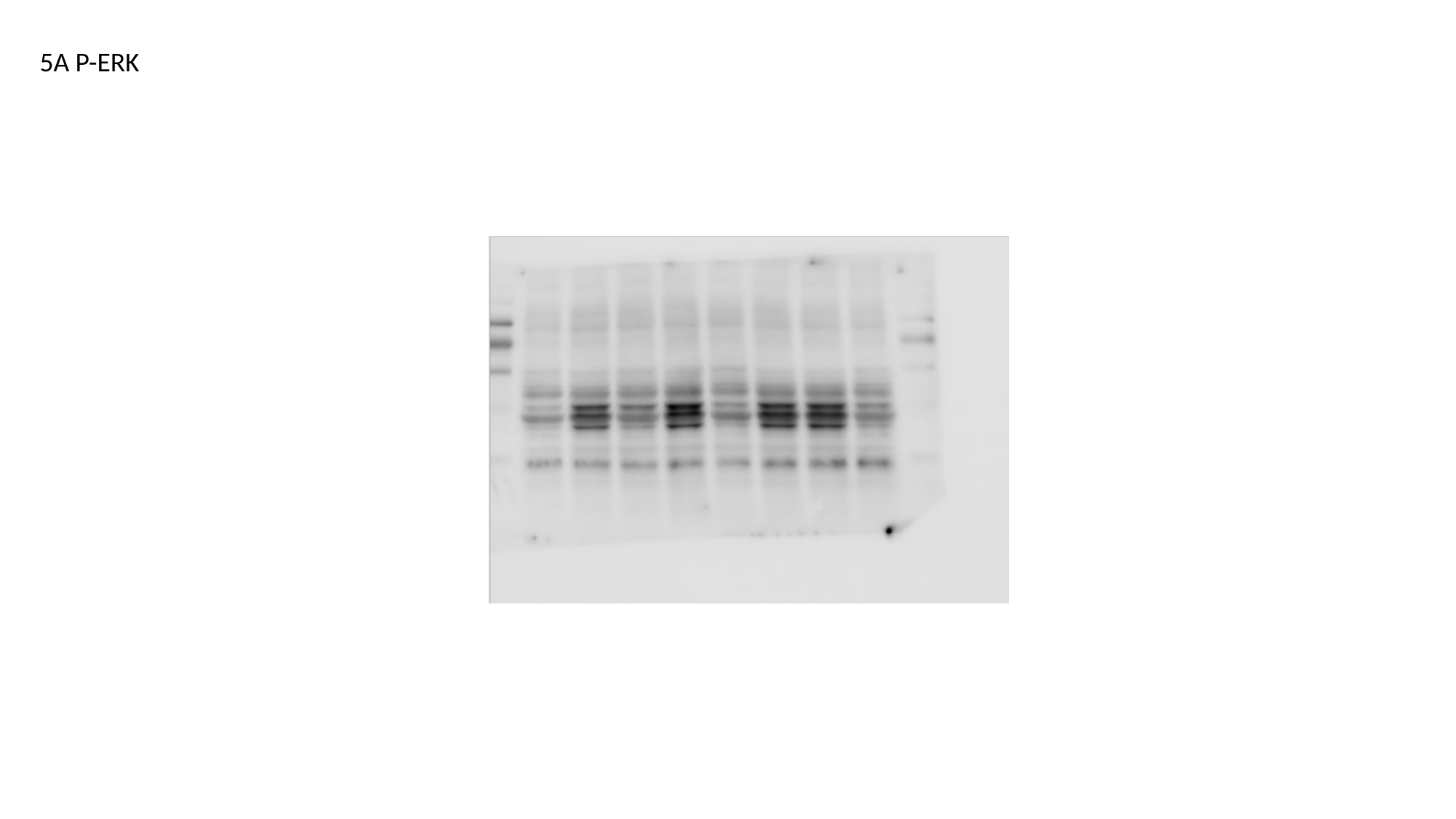

5A P-ERK

## Slide 4
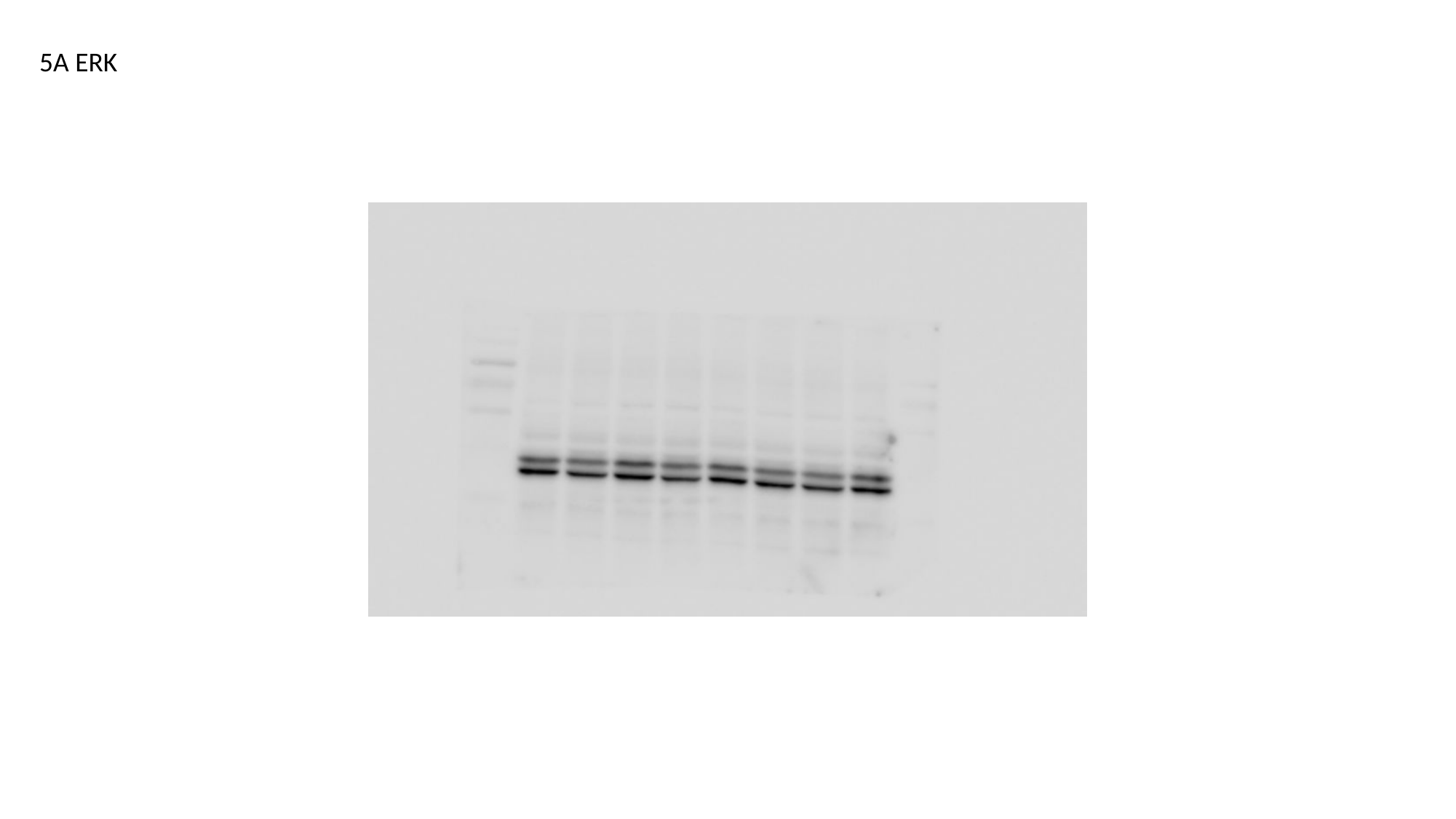

5A ERK

## Slide 5
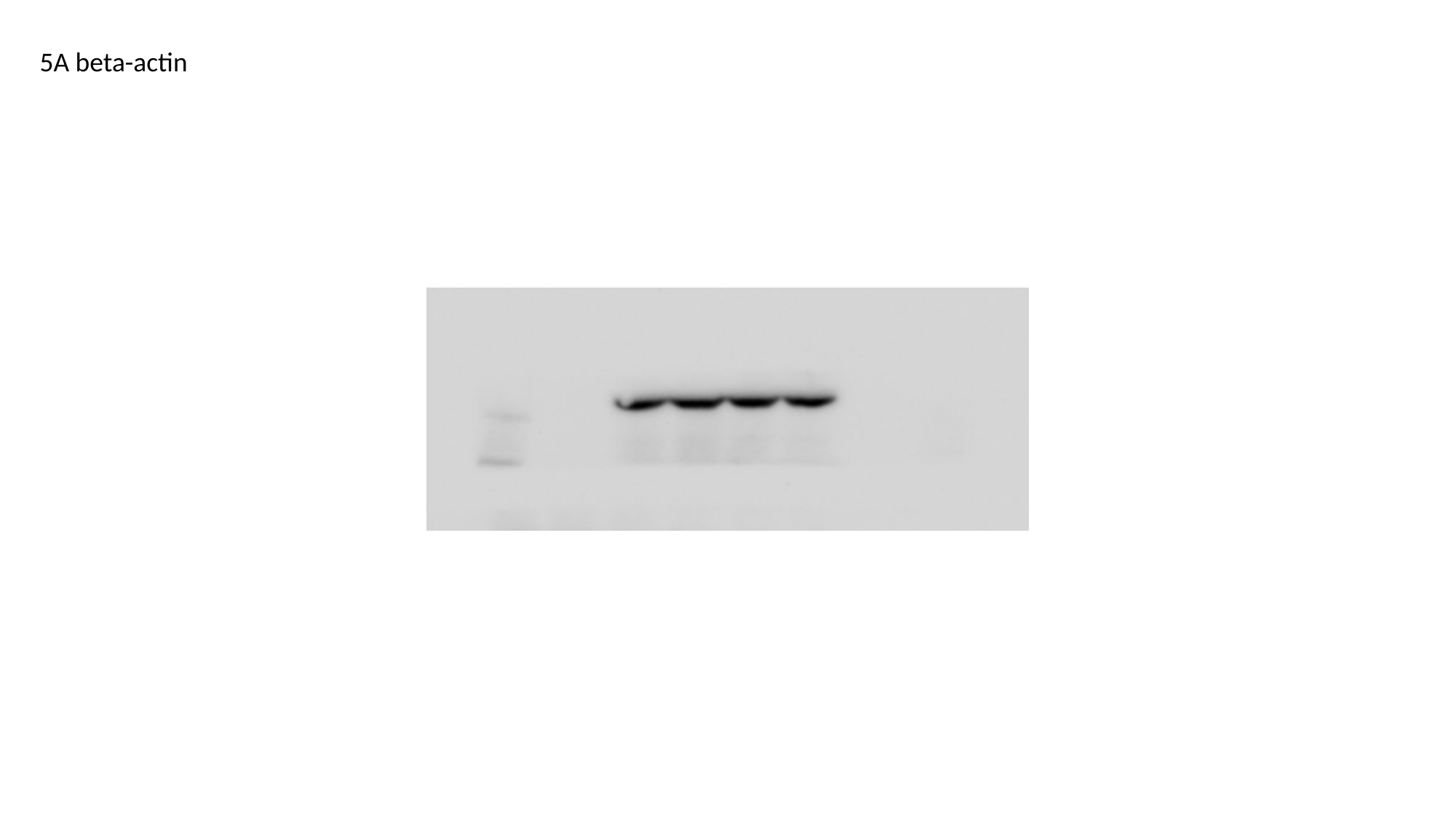

5A beta-actin

## Slide 6
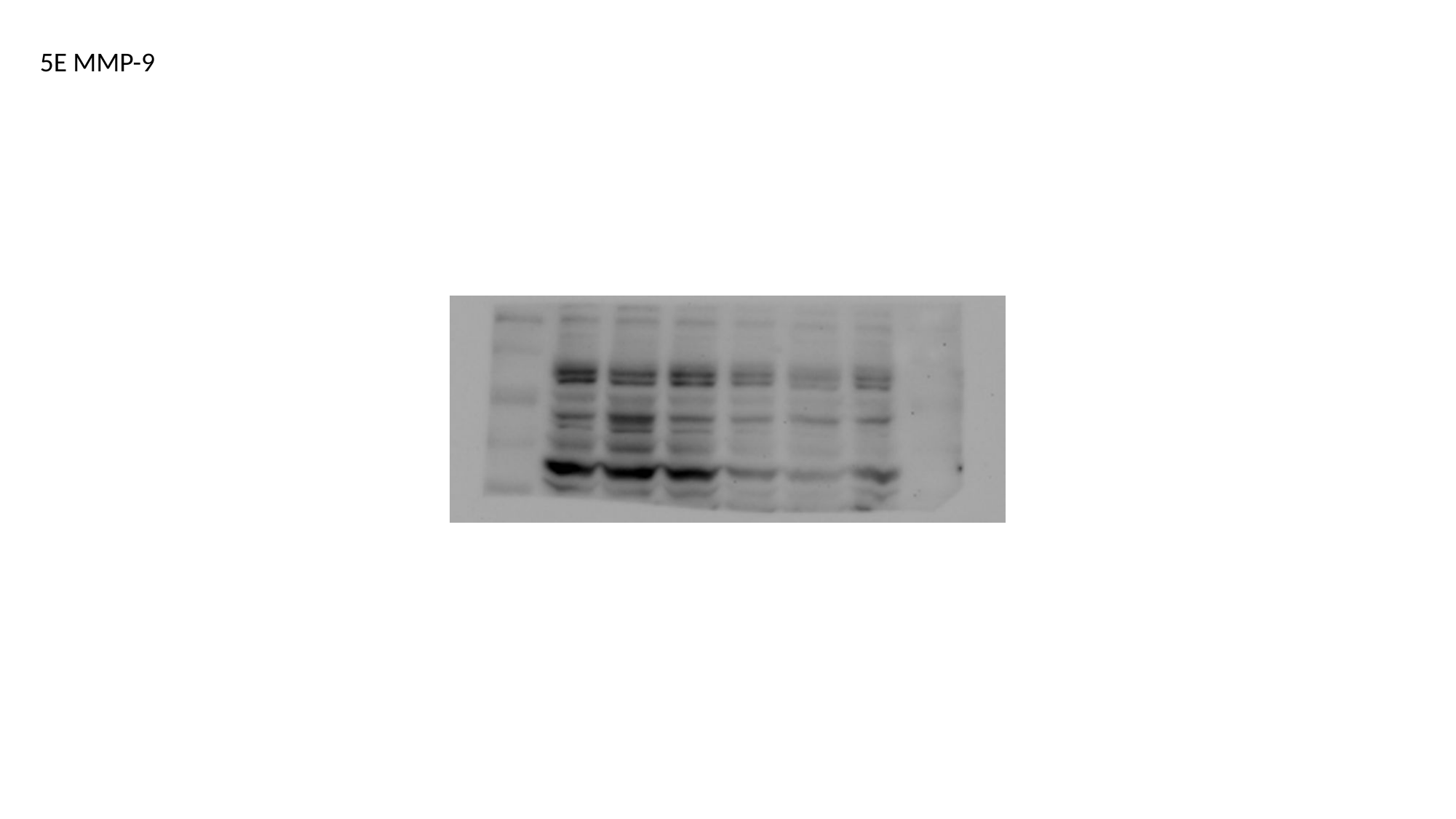

5E MMP-9

## Slide 7
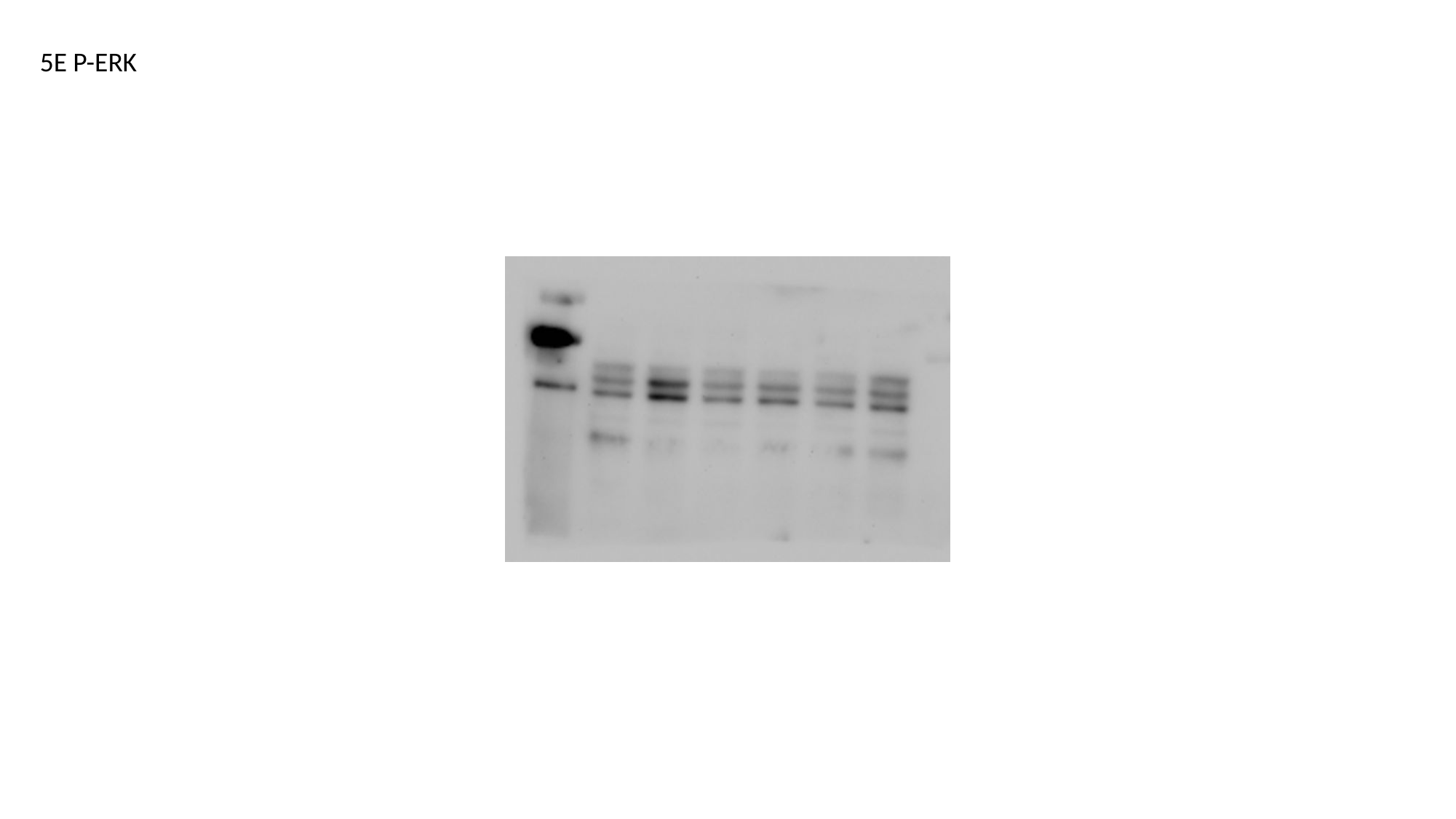

5E P-ERK

## Slide 8
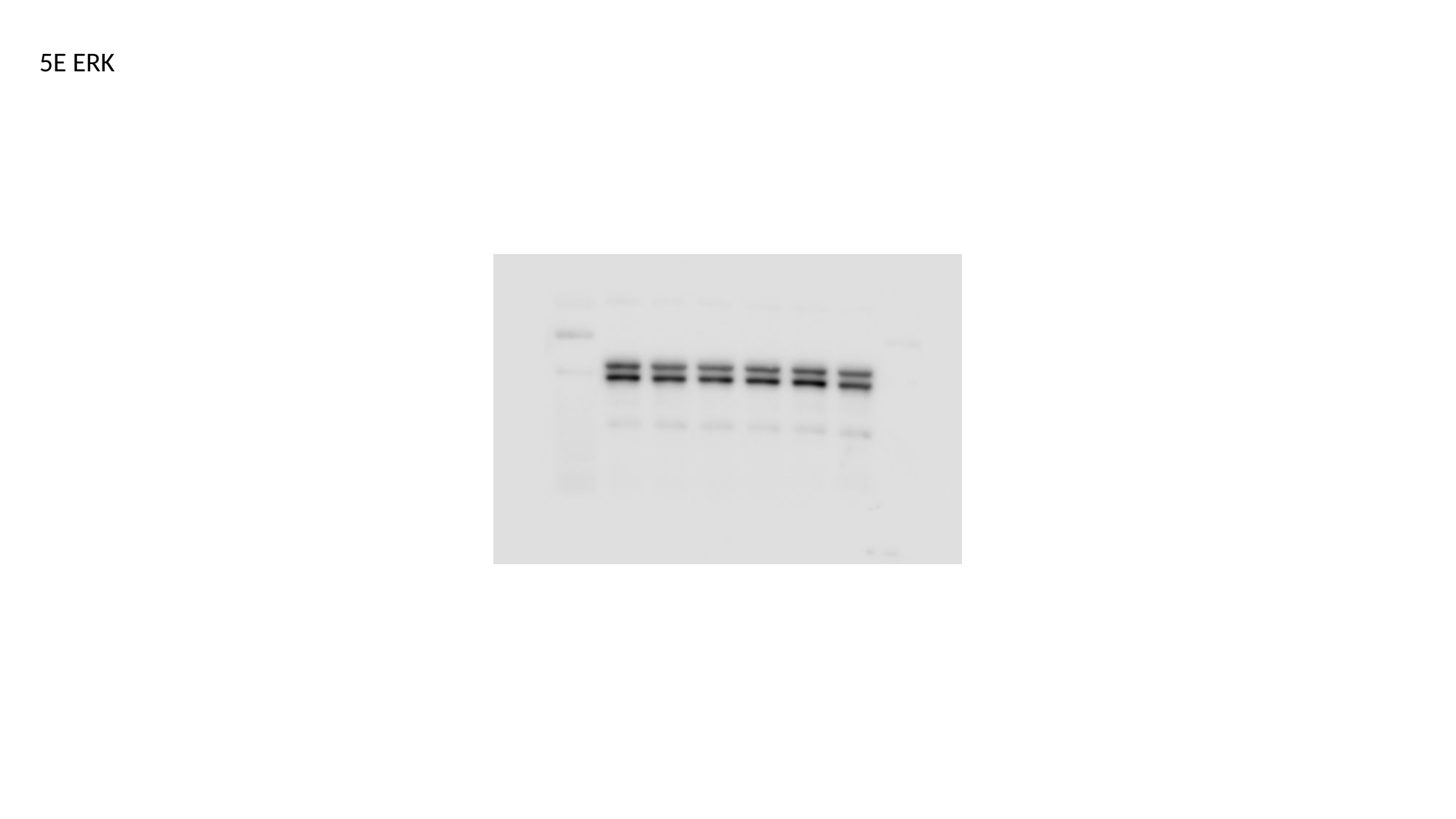

5E ERK

## Slide 9
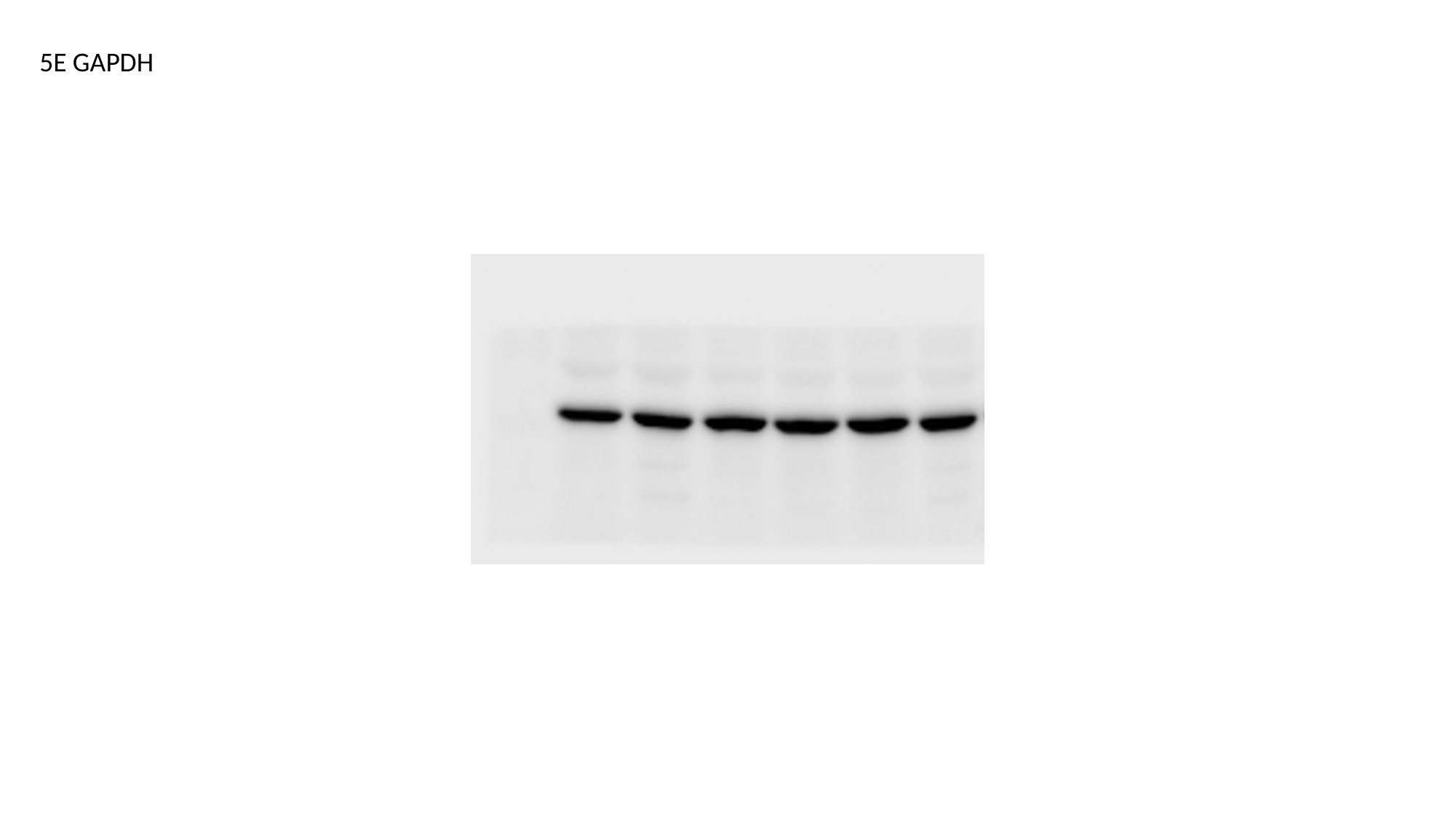

5E GAPDH

## Slide 10
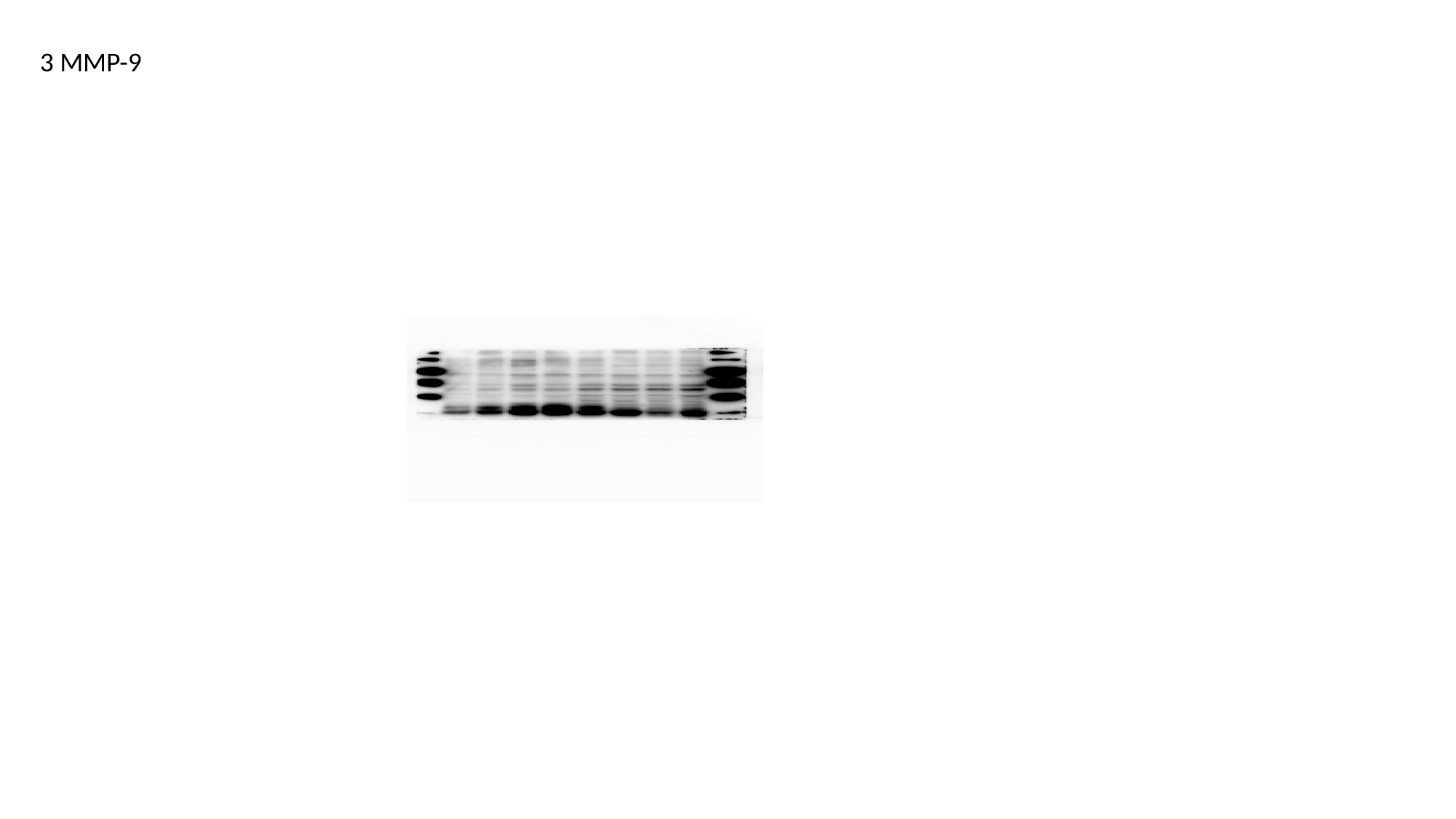

3 MMP-9

## Slide 11
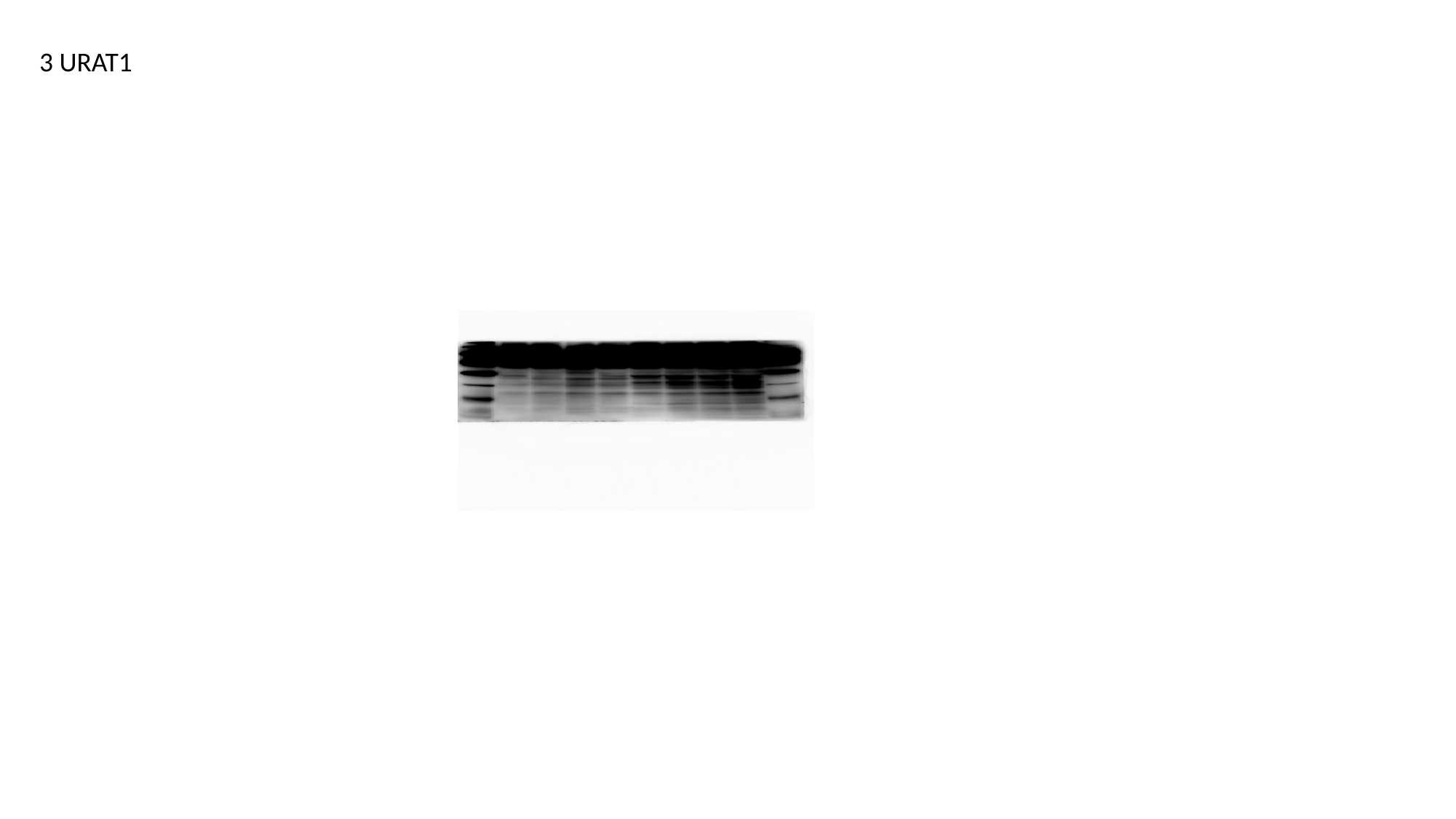

3 URAT1

## Slide 12
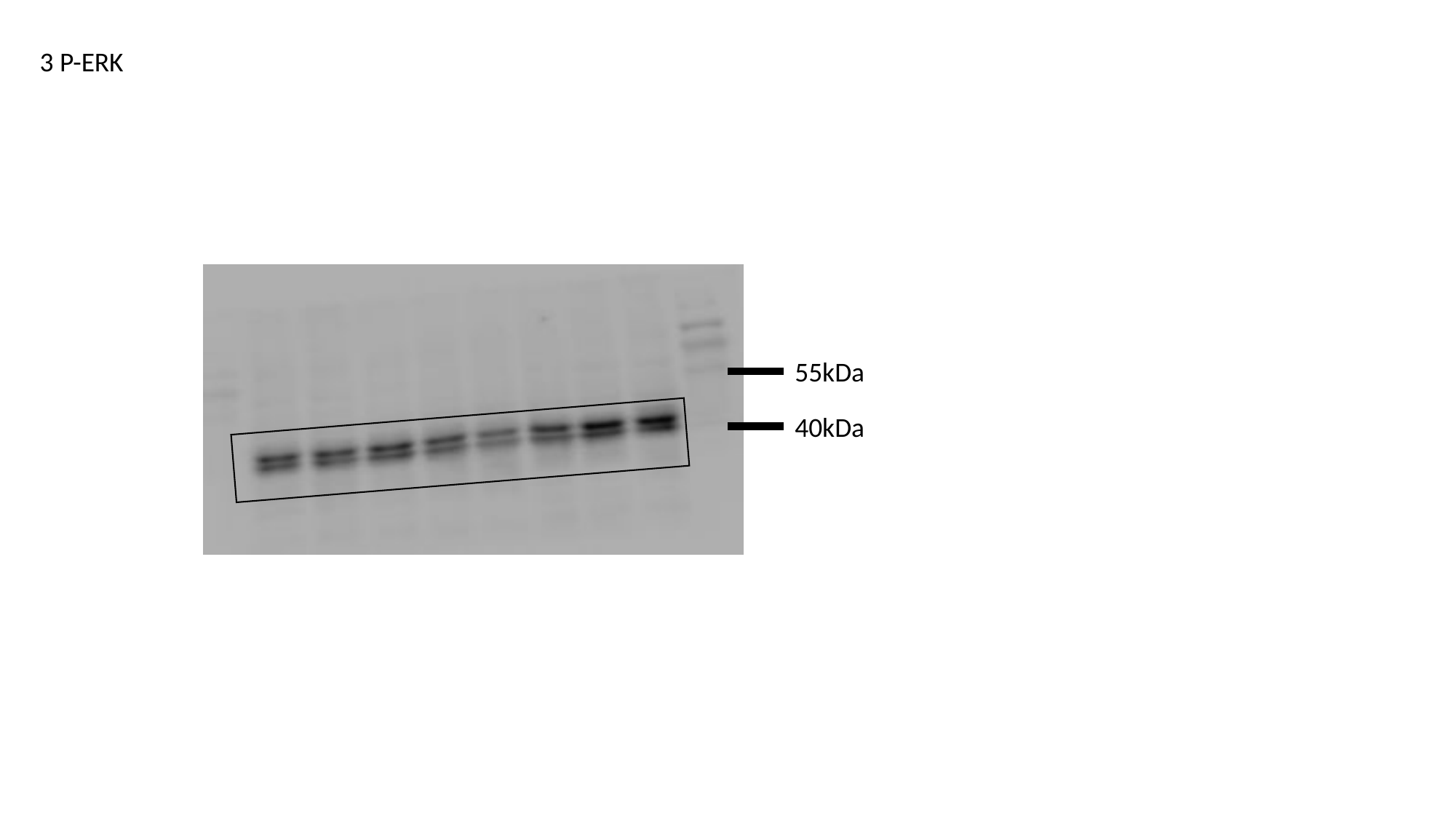

3 P-ERK
55kDa
40kDa

## Slide 13
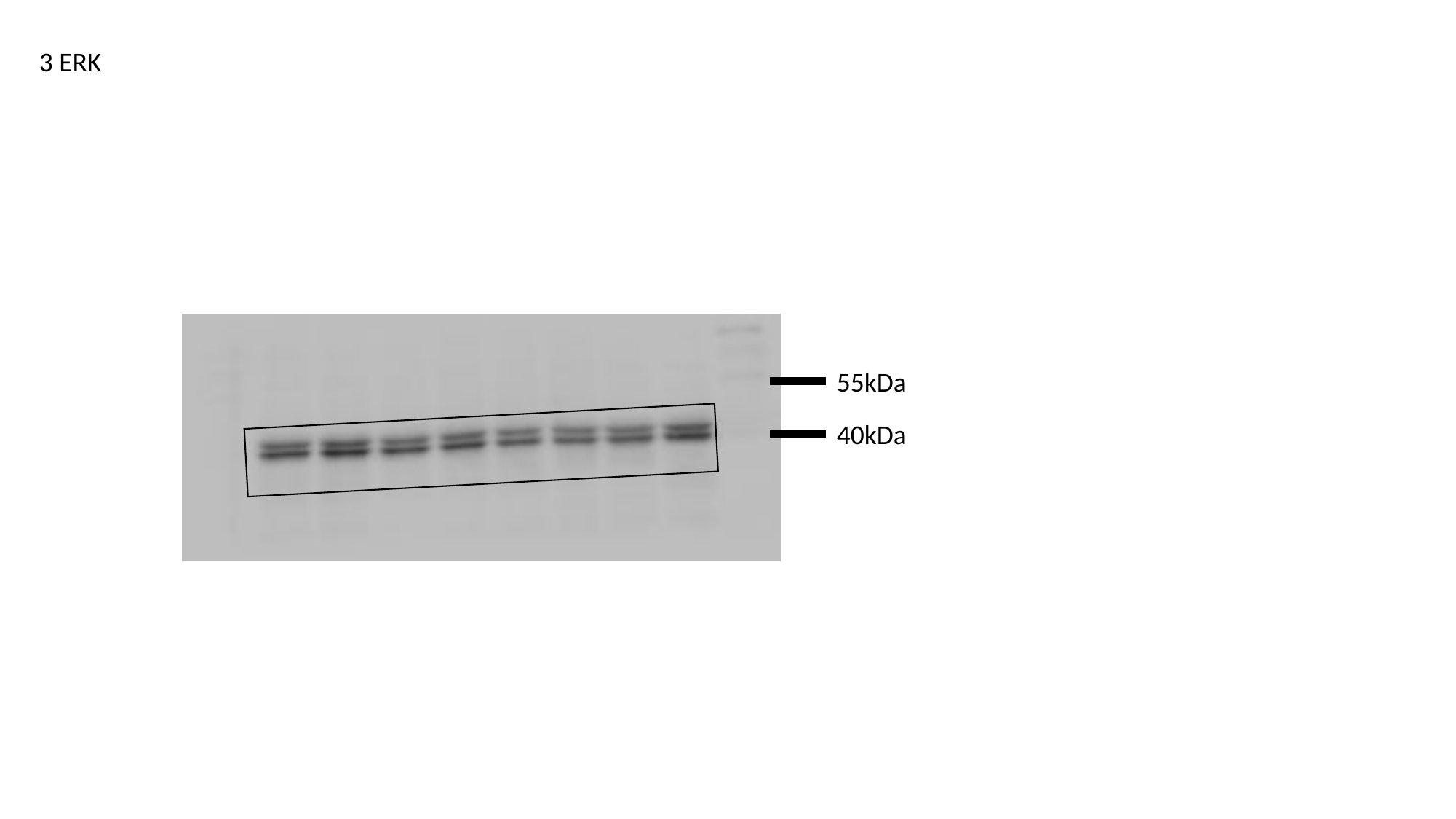

3 ERK
55kDa
40kDa

## Slide 14
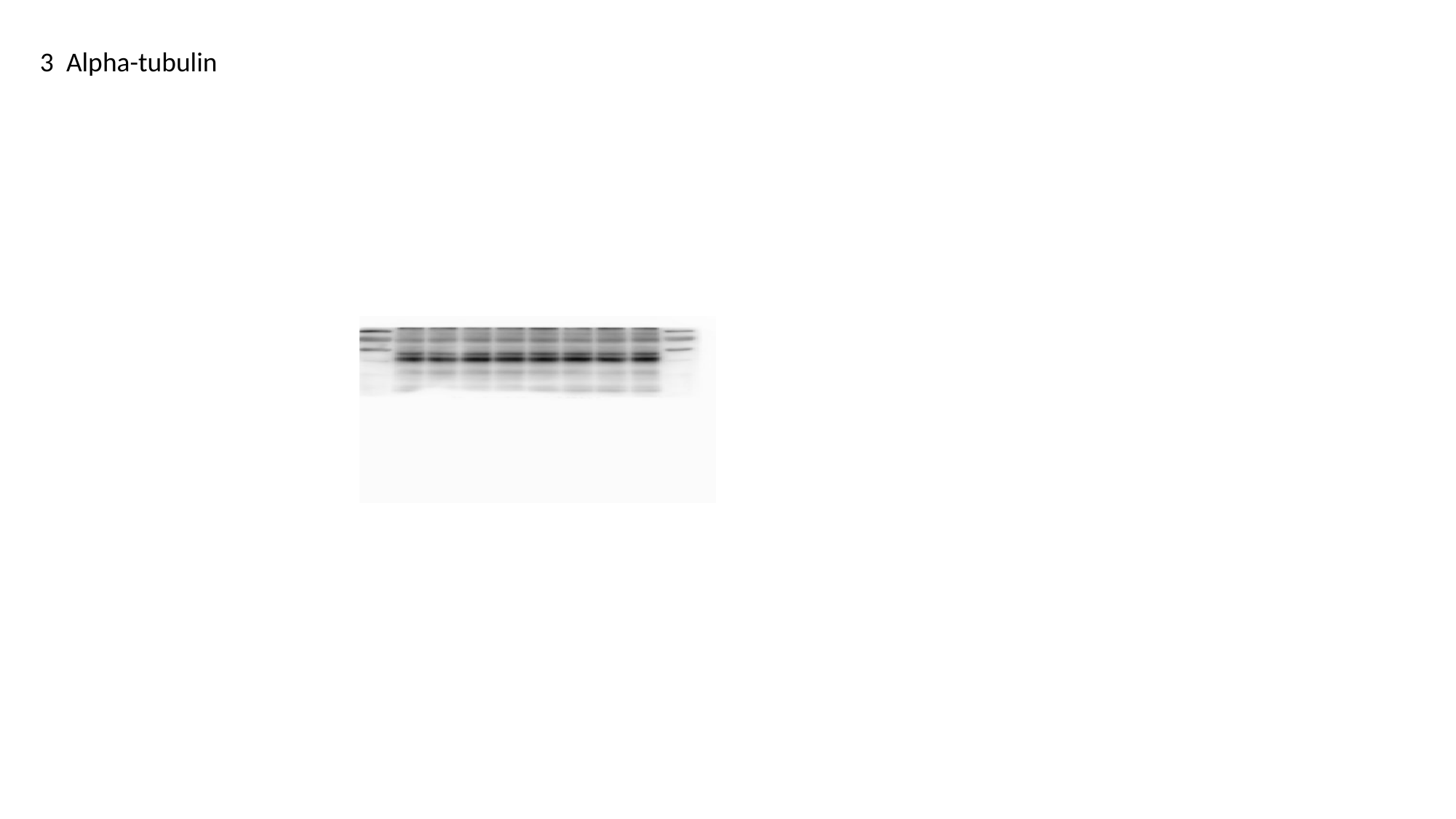

3 Alpha-tubulin
